# Two-Winged *Cloeodes* in Brazil: New Species, Stage Description, and Key to South American Species

**DOI:** 10.1673/031.013.6001

**Published:** 2013-06-22

**Authors:** Fabiana Criste Massariol, Lucas Ramos Costa Lima, Ulisses Dos Santos Pinheiro, Luciano Lopes Quieroz, Leandro Gonçalves Oliveira, Frederico Falcão Salles

**Affiliations:** 1 Laboratório de Sistemática e Ecologia de Insetos, Programa de Pós-graduação em Biodiversidade Tropical, Univer-sidade Federal do Espírito Santo, CEP 29.933-415, São Mateus, ES, Brazil; 2 Laboratório de Porifera, Programa de Pós-graduação em Biologia Animal, Universidade Federal de Pernambuco, CEP 50.670-420, Recife, PE, Brazil; 3 Laboratório de Porifera, Depto. de Zoologia, Universidade Federal de Pernambuco, CEP 50.670-420, Recife, PE, Brazil; 4 Laboratório de Análise e Gerenciamento Ambiental de Recursos Hídricos, Universidade Federal de Goiás, CEP: 74.001-970, Goiânia, GO, Brazil; 5 Laboratório de Análise e Gerenciamento Ambiental de Recursos Hídricos, Depto. de Ecologia, Universidade Federal de Goiás, CEP: 74.001 -970, Goiânia, GO, Brazil; 6 Laboratório de Sistemática e Ecologia de Insetos, Depto. de Ciências Agrárias e Biológicas, Universidade Federal do Espírito Santo, CEP 29.933-415, São Mateus, ES, Brazil

**Keywords:** macroinvertebrate, mayfly, Neotropics, new taxon, taxonomy

## Abstract

The present work, based on material from northern, central-western, and northeastern Brazil, contributes to the knowledge of the two-winged *Cloeodes* Traver (Ephemeroptera: Baetidae) in South America. Two new species, *C. maracatu,* sp. nov. and *C. spaceki,* sp. nov., are described, the former based on nymphs and reared adults and the latter only on nymphs; the male and female imago of *C. auwe* and the female imago of *C. redactus* are described. Based on these findings, an updated key for South American nymphs and male adults of the two-winged *Cloeodes* is provided.

## Introduction

The genus *Cloeodes* Traver (Ephemeroptera: Baetidae) is represented by approximately 35 species distributed in the Neotropical, Nearctic, Afrotropical, and Oriental regions (Traver 1938; Waltz and McCafferty 1994; Lugo-Ortiz et al. 1999; Soldán and Yang 2003; Jacobus et al. 2006). The genus is most diverse in the Neotropics, where 22 species have been described so far, including 20 from South America and six from the Antilles, Central America, and southern North America (Waltz and McCafferty 1987a, b; Kluge 1991; Hofman, Sartori, and Tomas 1999; Wiersema and Baumgardner 2000; Nieto and Richard 2008; Nieto and Emmerich 2011; Salles 2011).

All species of *Cloeodes* outside South America are two-winged (i.e., their hind wings or hind wings pads are absent), but this rule does not apply to South America. Of the 20 species known prior to this study, only six were two-winged: *C. anduzei* Traver, *C. auwe* Salles and Batista, *C. barituensis* Nieto and Richard, *C. binocularis* Needham and Murphy, *C. redactus* Waltz and McCaffety, and *C.* *turbinops* Needham and Murphy. *Cloeodes anduzei, C. binocularis,* and *C. turbinops* were described based only on male imagines, *C. barituensis* was described based on all stages, and *C. auwe* and *C. redactus* are known exclusively at the nymphal stage.

The present study, based on material from several localities in Brazil, contributes to the knowledge of the two-winged representatives of *Cloeodes*: two new species are described, one based on nymphs from central Brazil and the other based on nymphs and imagines from the Northeastern Region; the male and female imago of *C. auwe* and the female imago of *C. redactus* are described; furthermore, new diagnostic characteristics are presented for nymphs of both species. An updated taxonomic key for the South American species of this group is also proposed.

## Material and Methods

Taxonomic descriptions and/or diagnoses presented herein were generated from a DELTA (Dallwitz 1980; Dallwitz et al. 1993) database of South American Baetidae genera and species under development (e.g., Salles 2010; Massariol and Salles 2011). The free program DIVA-GIS 5.2 (http://www.diva-gis.org/) was used to make the distribution map of the species.

Photographs were taken using an OPTON Q719K-AC microscope with a TA-0124S digital camera, or a Leica M165C (http://www.leica-microsystems.com/) stereomicroscope with a DFC420 digital camera. In the latter case, a series of stacked images were processed with the program Leica Application Suite version 3.4.1 to produce final images with enhanced depth of field. Line drawings were made with the aid of a camera lucida, and/or photographs were prepared according to Coleman (2003, 2006).

The material examined is deposited in the following institutions: Invertebrate Collection of the Instituto Nacional de Pesquisas da Amazônia (INPA), Manaus, Brazil; Coleção Zoológica Norte Capixaba (CZNC), Universidade Federal do Espírito Santo, São Mateus, Brazil; Coleção Entomológica Prof. José Alfredo Pinheiro Dutra (DZRJ), Departamento de Zoologia, Universidade Federal do Rio de Janeiro, Rio de Janeiro, Brazil; and Instituto Miguel Lillo (IML), Tucumán, Argentina.

### Taxonomy

**Figure 1.**
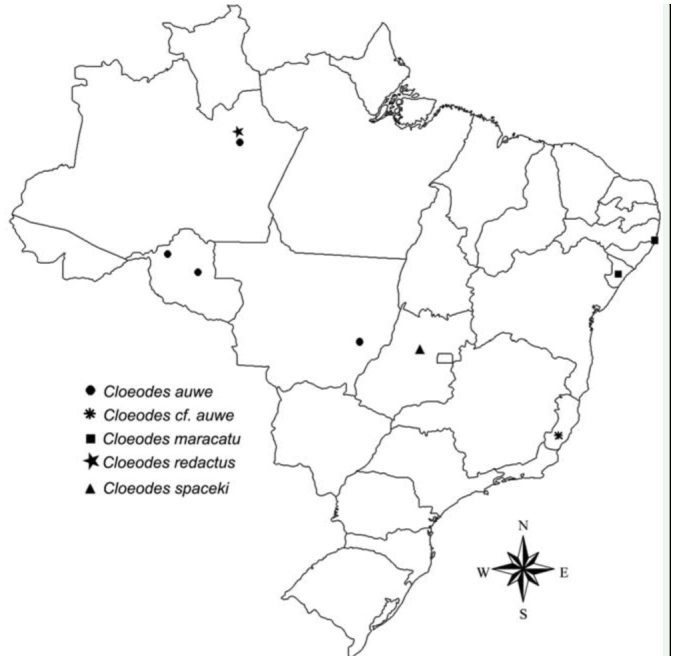
Distribution of the species of two-winged *Cloeodes* in Brazil. High quality figures are available online.

**Figure 2–4.**
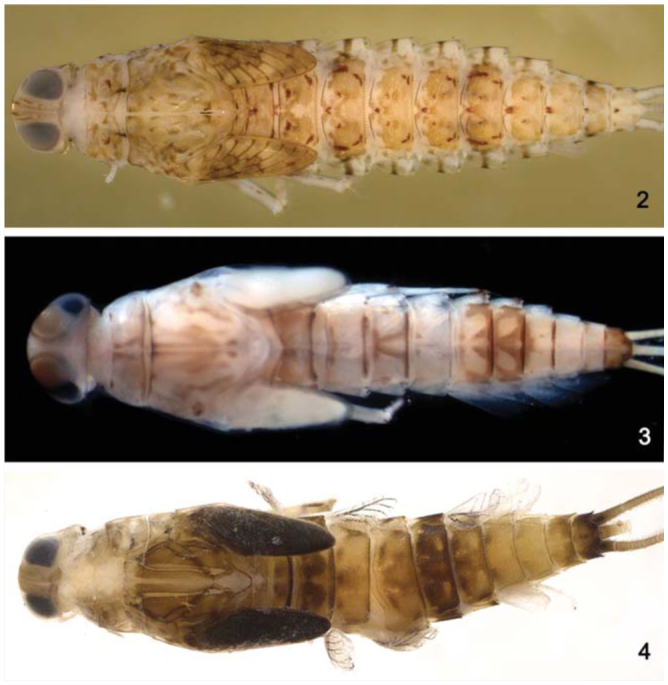
*Cloeodes* spp., nymphs in dorsal view. 2. *C. auwe.* 3. *C. barituensis.* 4. *C. maracatu*, sp. nov. High quality figures are available online.

**Figure 5–7.**
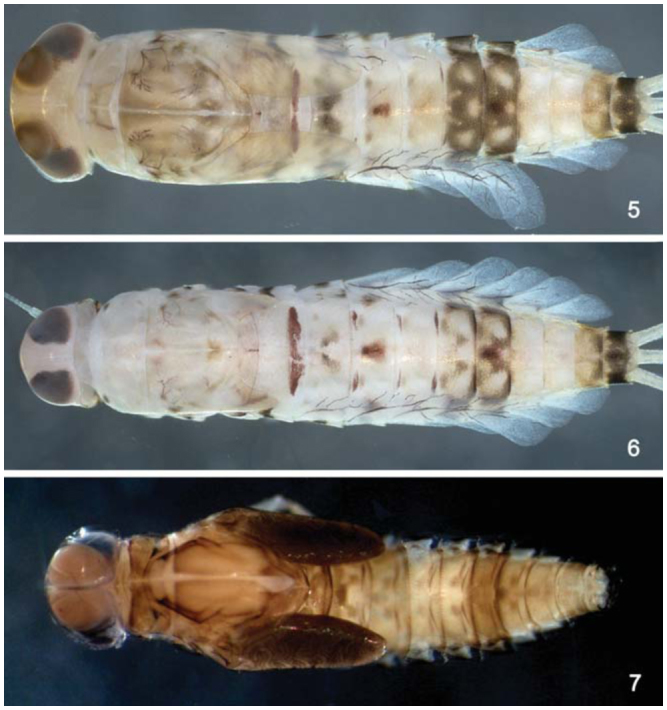
*Cloeodes* spp., nymphs in dorsal view. 5. *C. redactus*, male. 6. *C. redactus*, female. 7. *C. spaceki*, sp. nov. High quality figures are available online.

**Figure 8–10.**
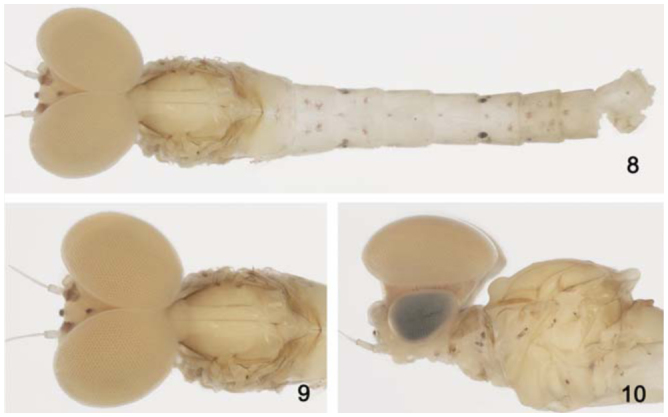
*Cloeodes auwe*, male adult. 8. Dorsal view. 9, 10. Detail of turbinate eyes and thorax, dorsal view and lateral view. High quality figures are available online.

**Figure 11–12.**
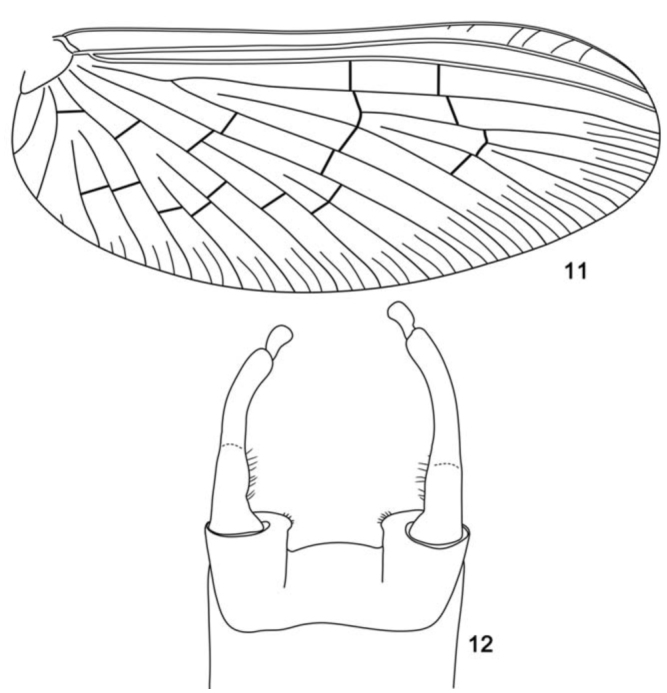
*Cloeodes auwe*, adult. 11. Fore-wing. 12. Male, genitalia. High quality figures are available online.

***Cloeodes auwe*Salles and Batista, 2004 (Figure 1, 2, 8–12)**
*Cloeodes auwe* Salles and Batista, in Salles et al. 2004: pp. 5; Domínguez et al. 2006: pp. 148.
**Diagnoses**
  **Nymphs** (Salles at al. 2004; Domínguez et al. 2006, adapted): 1) Antenna about 2.0× the length of head capsule; 2) Labrum with dorsal arc of setae composed of 1 (medial) + 0 (inbetween) + 2 (outer) long, spine-like setae; 3) Segment III of labial palp truncated; 4) Fore-femur with apex without projection, with two blunt setae; 5) Tarsal claw 0.5 to 0.8× length of tarsi; 6) General coloration of abdomen yellowish-white with light brown to dark brown irregular marks, segment I with a circular clear mark at anteromedial region; 7) Spines on posterior margin of tergum I present; 8) Paraproct with eight marginal spines; 9) Caudal filaments with posterior margin of segments with short spines on each segment and long spines on every four segments of cerci and terminal filament.
**Adults. Male imago:** 1) Turbinate eyes with inner margins divergent, touching each other posteriorly (Figure 8, 9); 2) Marginal intercalaries absent between Sc-R2 and CuP-A (Figure 11); 3) Abdominal terga VI with large macula on anterolateral regions (Figure 8); 4) Segment II of forceps with basal constriction (Figure 12); 5) Segment III of forceps elongated (Figure 12); 6) Posterior margin of subgenital plate slightly convex (Figure 12). **Female imago:** 1) General coloration yellowish-white with abdominal marks similar to male imago.
**Descriptions**
  **Male imago**
  **Length.** *Body:* 4.8 mm; *antenna:* 0.5 mm; *fore-wing:* 4.0 mm; *tibia I:* 0.8 mm; *tibia II:* 0.7 mm; *tibia III:* 0.7 mm; *caudal filament:* broken.
**Head** (Figure 8–10). Yellowish-white. Turbinate portion of compound eyes yellow dorsally, stalk reddish-brown. Antenna yellowish-white. Dorsal portion of turbinate eyes oblong; length 1.5× width; stalk height 0.4× width of dorsal portion; inner margins divergent, touching each other posteriorly (Figure 9).
**Thorax** (Figure 8–10). Yellowish-white. Mesonotum more opaque than pronotum and metanotum. Lateral margin of anteronotal and metascutellar protuberances brown. *Anteronotal protuberance* rounded (Figure 10).
*Metascutellar protuberance* posteriorly pointed (Figure 11).
*Legs* yellowish-white; femur with black transversal marks on posterior surface. Leg I: tibia 0.9× length of femur; tarsi 0.5× length of femur; and with four segments decreasing on length apically. Leg II: tibia 1.0× length of femur; tarsi 0.9× length of femur. Leg III: tibia 0.9× length of femur. Tarsus 0.9× length of femur.
*Fore-wing* (Figure 11) hyaline, but opaque between C and R1. Longitudinal veins light-brown, except C, Sc, and R1 yellowish-brown; cross veins dark brown; stigmatic area with four cross veins touching Sc and two veins not touching Sc; marginal intercalaries paired, except single between veins ICu2 and CuP, and absent between veins Sc and R2 and CuP and A; length of each intercalary vein 0.9× distance between adjacent longitudinal veins; length of fore-wing about 2.5× width. *Hind wing* absent.
**Abdomen** (Figure 8). *Terga* yellowish-white. Segment I–VI white, VII–X yellowish-white. Segment III–VIII and X with one red macula on anteromedial region. Segment II, III, VI, and VII with one red macula on postero sub-lateral regions. Segment II with one red macula on antero sublateral regions. Segment III with one red macula on anterolateral regions. Segment VI with one large black macula on anterolateral regions. Tracheation partially pigmented. *Sterna* yellowish-white.
*Genitalia* (Figure 12) yellowish-white. Forceps segment I sub-rectangular; 0.5× length of segment II; distance between base of forceps 1.2× distance between lateral margins of forceps. Forceps segment II with basal constriction. Forceps segment III elongated, 1.8× as long as wide; 0.2× length of segment II. Posterior margin of sub-genital plate slightly convex.
**Female imago**
  **Length.** *Body:* 5.2 mm; *antenna:* 0.5 mm; *fore-wing:* 4.8 mm; *tibia I:* 0.8 mm; *tibia II:* 0.7 mm; *tibia III:* 0.7 mm; *caudal filament:* broken.
**Head.** Yellowish-white. Compound eye black. Antenna yellowish-white.
**Thorax.** Yellowish-white.
*Anteronotal protuberance* rounded.
*Metascutellar protuberance* posteriorly pointed.
*Legs* yellowish-white; femur with black transversal marks on posterior surface. Leg I: tibia 0.9× length of femur; tarsi 1.0× length of femur; and with 4 segments decreasing in length apically. Leg II tibia 0.9× length of femur; tarsi 1.0× length of femur. Leg III tibia 0.9× length of femur; tarsi 1.0× length of femur.
*Fore-wing* hyaline, but opaque between C and R1. Longitudinal veins light brown, except C, Sc and R1 yellowish-brown; cross veins dark brown; stigmatic area with seven cross veins touching or almost touching Sc; marginal intercalaries paired, except single between veins ICu2 and CuP, and absent between veins Sc and R2, CuP and A; length of each intercalary vein 0.9× distance between adjacent longitudinal veins; length of fore-wing about 2.6× width.
**Abdomen.** *Terga* segments I–VI yellowish-white and VII–X white. Markings similar to male imago. Tracheation partially pigmented. *Sterna* yellowish-white.
**Comments**
  *Cloeodes auwe* is a very unusual member of the genus; the nymphs are unique among South American *Cloeodes* in several characteristics, such as shape of the third segment of labial palp and tarsal claws size. The very long marginal intercalary veins of the adults readily distinguish it from other species of *Cloeodes.* Although adults were not obtained by rearing, they are considered conspecific based on the abdominal color pattern of a mature nymph (Figure 2) collected at the same place where the adult was found. The species is a relatively common member of the mayfly community in Amazonian streams. However, among the material examined there was a small immature nymph from the Espírito Santo State, Southeastern Brazil. Given the unexpected presence of this species outside the Amazonas Basin, and the small size of the single nymph from Espírito Santo, this nymph was tentatively identified as *C. auwe.*
**Distribution** (Figure 1)
  BRAZIL - Amazonas, Mato Grosso, Rondônia.
**Material examined**
  One nymph, Brazil, Rondônia, Rio São Domingos, 30 km on the road to Tarilândia, 29 July 2004, 10° 37′ 46.9″ S, 62° 33′ 50.3″ W, 157 m a.s.l., Hamada N, col. Nine nymphs, Brazil, Rondônia, Rio Jaci, above do Rio São Pedro, 25 March 2004, 09° 31′ 21.0″ S, 64° 21′ 58.1″ W, Hamada N, col. Thirty-four nymphs, Brazil, Amazonas, Presidente Figueiredo, Igarapé do Km 24, 14 October 2003, 02° 45′ 51.9″ S, 60° 02′ 11.9″ W, rock, low current, Salles FF, col. One nymph, Brazil, Amazonas, Manaus, BR 174, Km 18.5, 16 July 2009, 02° 49′ 1.5″ S, 60° 02′ 7.5″ W, Salles FF, col. One male imago and two female imagines, Brazil, Amazonas, Manaus, BR 174, Km 18.5, 22 January 2009, 02° 49′ 1.5″ S, 60° 02′ 7.5″ W, light trap, Salles FF, col. (CZNC).
*Cloeodes cf. auwe.* One nymph, Brazil, Espírito Santo, Afonso Cláudio, Cachoeira Santa Luzia, 15 February 2011, 20° 09′ 33.2″ S, 41° 08′ 53.8″ W, 457 m a.s.l., rock, low current, Massariol FC, Bertazo K, col. (CZNC).
**Life cycle association**
  Although adults were not obtained by rearing, they are considered to be conspecific based on the abdominal color pattern of a mature nymph collected at the same place where the adults were collected.

**Figure 13–18.**
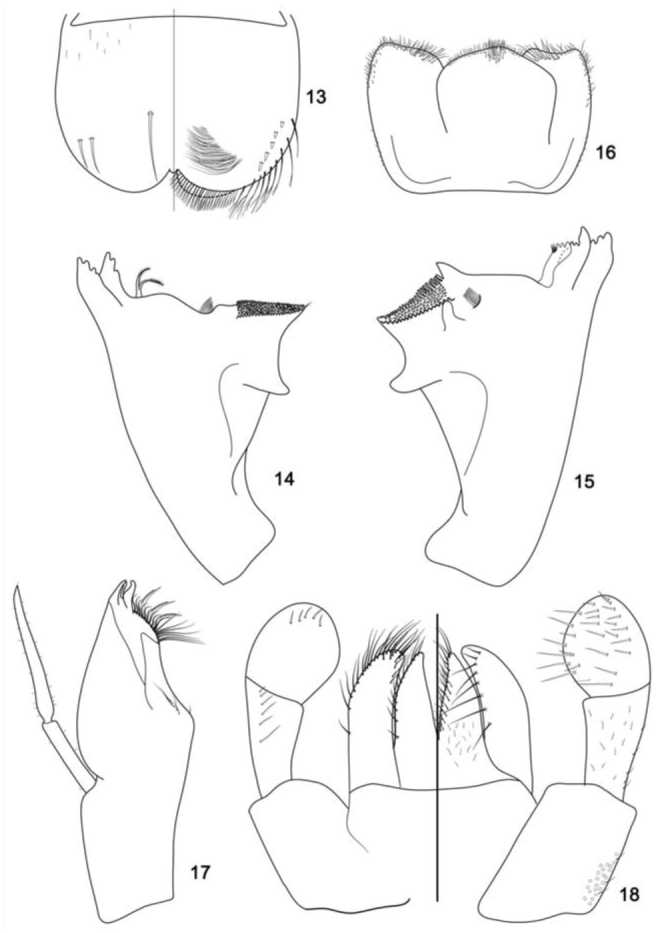
*Cloeodes maracatu*, sp. nov., nymph. 13. Labrum (left dorsal view, right ventral view). 14. Right mandible, ventral view. 15. Left mandible, dorsal view. 16. Hypopharynx, dorsal view. 17. Maxilla, ventral view. 18. Labium (left, dorsal view; right, ventral view.). High quality figures are available online.

**Figure 19–23.**
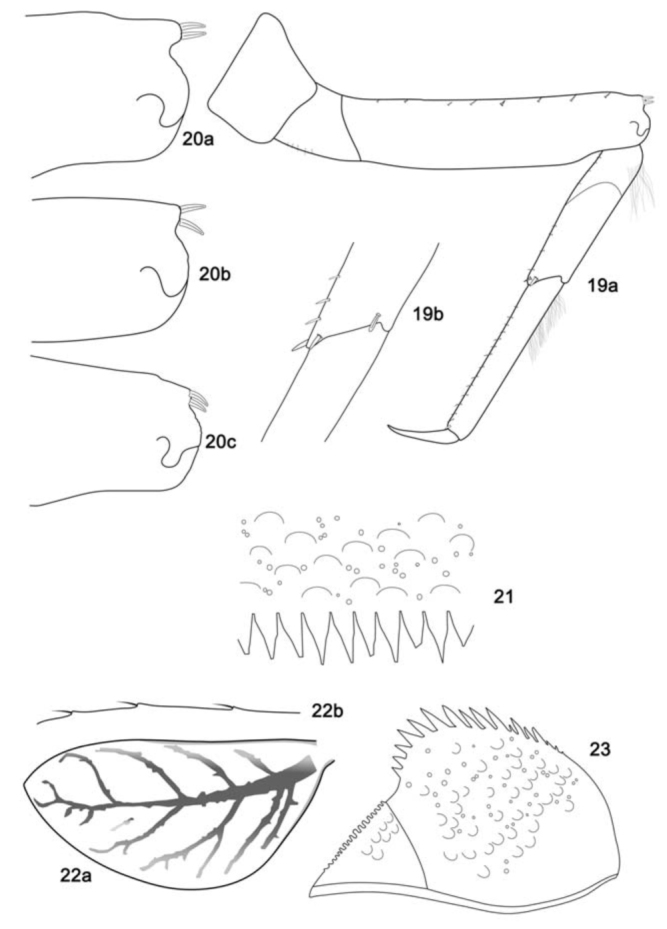
*Cloeodes maracatu*, sp. nov., nymph. 19. Foreleg, anterior surface (a: general view; b: sub-tending bristle). 20. Projections of femur apex (a: fore-femur; b: mid-femur; c: hind-femur). 21. Posterior margin of tergum IV. 22. Gill IV (a: general view; b: details of gill margin). 23. Paraproct (dorsal view). High quality figures are available online.

**Figure 24–27.**
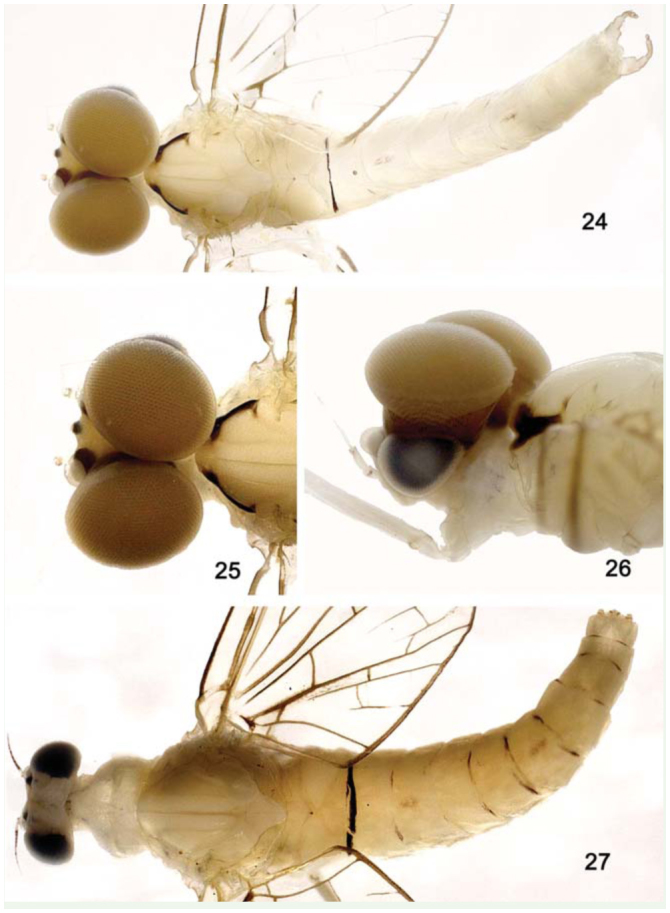
*Cloeodes maracatu*, sp. nov., adults. 24. Male, dorsal view. 25, 26. Male, detail of turbinate eyes and thorax, dorsal view, lateral view. 27. Female, dorsal view. High quality figures are available online.

**Figure 28–30.**
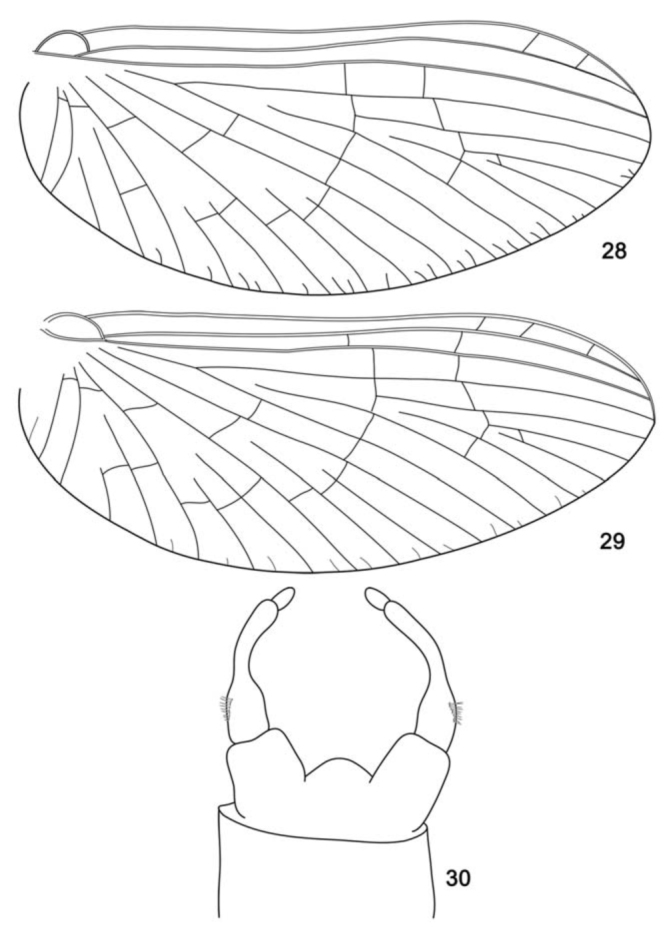
*Cloeodes maracatu*, sp. nov, adults. 28. Male, fore-wing. 29. Female, fore-wing. 30. Male, genitalia. High quality figures are available online.

***Cloeodes maracatu* Lima, Pinheiro and Massariol, sp. nov. (Figure 1, 4, 13–30)**
**Diagnoses**
  **Nymphs:** 1) Antenna about 2.2× length of head capsule; 2) Labrum with dorsal arc of setae composed of 1+0+2 long, spine-like setae (Figure 13); 3) Segment III of labial palp rounded (Figure 18); 4) Fore femur with apex projected, with 2 blunt setae (Figure 20a); 5) Tarsal claw 0.4× length of tarsi; 6) General coloration of abdomen yellowish-brown, segments I, II, VI, and IX washed with brown and VI and X widely washed with dark-brown (Figure 4); 7) Spines on posterior margin of tergum I present; 8) Paraproct with 15 to 18 marginal spines (Figure 23); 9) Caudal filaments with posterior margin of segments with short spines on each segment and long spines on every two segments on cercus and every four segments on terminal filament.
**Adults: Male imago.** 1) Turbinate eyes with inner margins divergent (Figure 25); 2) Marginal intercalaries paired, absent between Sc and R1 and CuP and A (Figure 28); 3) Abdominal terga I–VII with a narrow brown marks on anterolateral margin; 4) Segment II of forceps with basal constriction (Figure 30); 5) Segment III of forceps elongated (Figure 30); 6) Posterior margin of subgenital plate rounded (Figure 30). **Female imago.** 1) Marginal intercalaries single, and absent between Sc and R1, R sector, and CuP and A (Figure 29; 2) Abdominal terga yellowish-white, except for segments VII–X translucent white; posterior margin of segment I with a narrow transverse dark brown (Figure 27);
**Descriptions**
  **Nymph**
  **Length.** *Body:* 4.8–5.9 mm; *cercus:* 1.7–1.9 mm; *terminal filament:* 1.5–1.6 mm; *antenna:* 1.6–2.0 mm.
**Head** (Figure 4). Light brown with a narrow, pale-yellow median longitudinal band along the length of vertex; compound eyes and ocellus of the male side surrounded in black. Frons with the area between the bases of antenna yellow. Turbinate portion of compound eyes of the male reddish-brown.
*Antenna* light brown; 2.2× length of head capsule.
*Labrum* (Figure 13). Rectangular, broader than long, length about 0.7× maximum width; dorsal surface flat; distal margin with medial emargination and small process. Dorsally with many short, fine, simple setae scattered over surface; dorsal arc of setae composed of 1 + 0 + 2 long, spine-like setae; lateral margin bare. Ventrally with submarginal row of setae decreasing in length toward medial region, composed of lateral and anterolateral bifid and frayed setae, medial setae pectinate and bifid; ventral surface with five to eight short, blunt setae near the lateral and anterolateral margins.
*Right mandible* (Figure 14). Inner and outer set of incisors with three and four denticles respectively. Prostheca slender, bifurcated at middle, inner lobe long, outer short, both frayed. Margin between prostheca and mola slightly convex; tuft of setae between prostheca and mola absent; tuft of spine-like setae at base of mola present; tuft of setae at apex of mola present, reduced to a bifid setae. Lateral margins almost straight; bare; basal half bare.
*Left mandible* (Figure 15). Inner and outer set of incisors with four denticles respectively. Prostheca robust, apically denticulate and with comb-shape structure at apex. Margin between prostheca and mola slightly convex, without crenulations; tuft of setae absent; tuft of spine-like setae at base of mola present; subtriangular process wide, above level of area between prostheca and mola; denticles of mola not constricted; tuft of setae at apex of mola absent. Lateral margins almost straight; bare; basal half bare.
*Hypopharynx* (Figure 16). Lingua subequal in length to superlingua; apex with anteromedial lobe rounded, with short, fine, simple setae; medial tuft of short setae present; distal half laterally expanded. Superlingua not expanded; fine, simple setae scattered over lateral and distal margin and basal half of lateral margin with short, spine-like setae.
*Maxilla* (Figure 17). Crown of galea-lacinia with four denticles, inner denticle opposed to outer denticles; inner dorsal row of setae with three pectinate denti-setae. Medial protuberance of galea with one short, spine-like setae and five to six long setae. Maxillary palp reaching apex of galea-lacinia; two-segmented; setae on maxillary palp, short, fine and simple, scattered over surface, a spine-like setae at apex of segment II; palp segment II 1.8× length of segment I; apex of last segment constricted at base.
*Labium* (Figure 18). Glossa basally broad, narrowing apically and subequal in length to paraglossa; inner margin with 18 to 19 spinelike setae increasing in length apically, outer margin with 12 to 14 long, spine-like setae increasing in length apically; ventral surface with a row of seven to eight long and spinelike setae near inner margin and short, fine, simple setae scattered on basal half. Paraglossa sub-rectangular or straight, curved only at apex; apex with two rows of spine-like setae; outer margin with a row of 15 to 17 long and spine-like setae; dorsally and ventrally with a curved row of six to eight spine-like setae near to inner margin. Labial palp with segment I 0.8× length of segments II and III combined; segment I covered with short, simple setae and micropores near to outer margin; inner margin of segment II bare; outer margin with short, fine setae; dorsally with row of six to seven spine-like, simple setae; ventrally with short, fine, simple setae scattered over surface; segment III rounded; length 1.0× width; covered with spine-like simple setae along margins and ventral surface; dorsally with a row of spine-like simple setae near to apex.
**Thorax** (Figure 4). General coloration brown with yellow marks. Fore wing pads brownish.
*Hind wing* pads absent.
*Fore-leg* (Figure 19a). Brown, tarsi and tarsal claw yellowish-brown. Ratio of fore-leg 2.0 : 1.0 :0.5 : 1.2 :0.4 mm.
*Fore-femur.* Length about 4.5× maximum width; dorsally with a row of seven to nine blunt setae (in lateral view they look like spine-like setae); length of setae about 0.1× maximum width of femur; apex projected; with two blunt setae (Figure 20a); ventrally bare; anterior surface with abundant scale-bases and scales near to ventral margin.
*Tibia.* Dorsally with row of abundant, long, fine, simple setae; ventrally with row of 10 short, spine-like setae, lanceolate setae subapically; anterior surface with abundant scale-bases and scales near to ventral margin; tibio-patelar suture present. Subtending bristle present (Figure 19b).
*Tarsus.* Dorsally with row of abundant long, fine setae; ventrally with row of 15 to 17 spine-like setae and one long lanceolate setae near the apex; anterior surface with scalebases and scales scattered over surface; tarsal claw bare, 0.4× length of tarsi.
*Mid- and hind-leg differences.* Similar to foreleg, except for: subapical projections of femur less developed on mid-leg and practically absent on hind-leg (Figure 20a, b, c).
**Abdomen** (Figure 4). General coloration yellowish-brown washed with brown to dark brown. Segments I, II, VI, and IX washed with brown and VI and X widely washed with dark brown. Segments I–VI and X with yellow spots, segments VII–IX clearer; males showing segments III–IV clearer.
*Terga.* Surface with abundant scale bases and micropores; posterior margin of terga with regular spines, 1.9× as long as wide (Figure 21). Spines present in posterior margin of segments I–X.
*Sterna.* Spines present in posterior margin of segments III–IX.
*Gill* (Figure 22a, b). Opaque, trachea dark gray; inner and outer margins brown. Margin with broad spines and short, fine, simple setae (Figure 22b). Tracheae extending from main trunk to inner and outer margins. Gill I about 1.6× length of segment II; oval. Gill IV as long as the length of the segment V and VI of the combined half; oval. Gill VII about 2.0× length of segment VIII, oblong.
*Paraproct* (Figure 23). With 15 to 18 marginal spines; surface with abundant scale-bases and micropores; posterolateral extension with blunt marginal spines and scale-bases scattered on surface.
*Caudal filaments.* Yellowish-brown. Posterior margin of segments with short spines on each segment, and long spines on every two segments on cercus and every four segments on terminal filament; inner margin of cercus and inner and outer margin of terminal filament with tufts of long, flat setae.
**Male imago**
  **Length.** *Body:* 5.0 mm; *antenna:* 1.1 mm; *fore-wing:* 4.3 mm; *tibia I:* 1.4 mm; *tibia II:* 1.0 mm; *tibia III:* 0.9 mm; *caudal filament:* 10.2 mm.
**Head** (Figure 24–26). Yellowish brown. Turbinate portion of compound eye dorsally orange brown, stalk reddish brown. Antenna pale-yellow.
Dorsal portion of turbinate eye oblong; length 1.3× width; stalk height 0.8× length of dorsal portion; inner margins divergent (Figure 25).
**Thorax** (Figure 24, 26). General coloration pale-yellow. Medioscutelum with diagonal dark brown marks. Metanotum with grayish margins.
*Anteronotal protuberance* rounded.
*Metaescutelar protuberance* pointed and projected dorsally.
*Legs.* General coloration translucent white; femur with subapical dark mark and tibia with black longitudinal mark. Leg I: tibia 1.3× length of femur; tarsi 1.4× length of femur and four segments decreasing on length apically. Leg II: 1.2× tibia length of femur, tarsi 0.5× length of femur. Leg III: tibia 1.4× length of femur, tarsus 0.4× length of femur.
*Fore-wing* (Figure 28). Hyaline, except opaque between C and R1. Longitudinal and cross veins brown. Stigmatic area with four veins with two or three cross-veins touching subcostal vein. Marginal intercalaries paired, except single between veins IMA and MA2, IMP and MP2, CuA and ICu1, and absent between veins Sc and R1, R1 and R2, ICu1 and ICu2, ICu2 and CuP, and CuP and A; length of each intercalary vein 0.5× the distance between adjacent longitudinal veins; length of fore-wing about 3.0× the width.
*Hind wing* absent.
**Abdomen** (Figure 24). *Terga.* Segments I–VI translucent white and VII–X yellowish-white. Tergum I with black line on posterior margin. Segments I–VII with a narrow brown marking on anterolateral margin. Segments III and VII with brown longitudinal median marking. Tracheation not pigmented.
*Sterna.* Segments II–VI translucent, I and VII–X white. Caudal filaments pale-yellow, with black bases.
*Genitalia* (Figure 30). White. Forceps segment I sub-rectangular; 0.5× length of segment II; distance between the base of forceps 0.3× distance between the lateral margins of forceps. Forceps segment II with basal constriction. Forceps segment III elongated, 1.5× longer than wide, 0.2× length of article II. Posterior margin of subgenital plate rounded.
**Female imago**
  **Length.** *Body:* 4.8 mm; *antenna:* 0.8 mm; *fore-wing:* 4.0 mm; *tibia I:* 0.7 mm; *tibia II:* 1.0 mm; *tibia III:* 1.0 mm; *caudal filament:* 11.0 mm.
**Head** (Figure 27). Yellowishwhite. Compound eye black. Antenna with flagellum dark brown; scape and pedicel whitish, with brown apex.
**Thorax** (Figure 27). General coloration similar to imago except pronotum white and medioscutelum without diagonal dark brown marks.
*Anteronotal protuberance* rounded.
*Metaescutelar protuberance* pointed and projected dorsally.
*Legs.* Similar to male imago, except by tarsomeres of tibia I with longitudinal blackish mark. Leg I: tibia 0.9× length of femur, tarsi 0.7× length of femur. Leg II: tibia 1.1× length of femur, tarsus 0.3× length of femur. Leg III: tibia 1.1× length of femur, tarsus 0.3× the length of femur.
*Fore-wing* (Figure 29) hyaline, except area between C and R1 opaque. Longitudinal and transverse veins brown. Stigmatic area with three Sc veins touching or almost touching subcostal vein. Marginal intercalaries paired, except single between Sc and R1, and absent between R sector and CuP and A; length of intercalary vein 0.4× distance between adjacent longitudinal veins; length of fore-wing about 2.4× width.
*Hind wing* absent.
**Abdomen (Figure 27).** *Terga.* Yellowishwhite, except for segments VII–X translucent white. Posterior margin of segment I with a narrow transverse dark brown. Tracheation darkened. *Sterna.* Yellowish-white.
**Etymology**
  The specific epithet is an allusion to a popular musical style of Pernambuco State.
**Comments**
  Nymphs of *C. maracatu* are somewhat similar to those of *C. spaceki* sp. nov., however they can be easily differentiated based on the shape of the third segment of the labial palp (rounded in *C. maracatu,* truncated in *C. spaceki).* The adults of *C. maracatu,* as those of *C. barituensis,* present a sexual dimorphism related to the presence of single (female) or double (male) marginal intercalary veins. The body color pattern, especially the thorax, readily distinguishes *C. maracatu* from *C. barituensis.*
**Distribution** (Figure 1)
  BRAZIL - Pernambuco, Sergipe.
**Material examined**
  **Holotype.** Male imago with corresponding nymphal exuvia, Brazil, Pernambuco, Tamandaré, Reserva Biológica Saltinho, Rio Mamucabas, 21 September 2009, 08° 43′ 52.7″ S, 35° 10′ 24.8″ W, 38 m a.s.l., marginal vegetation, Lima LRC, col. (INPA).
**Paratypes.** One female imago, same data as holotype. One male imago, Brazil, Pernambuco, Rio Formoso, Riacho da Gameleira, 24 October 2009, 8° 43′ 12.3″ S, 35° 10′ 32.9″ W, 18 m a.s.l., rock, Lima LRC, col (CZNC). One female imago with corresponding nymphal exuvia, Brazil, Pernambuco, Tamandaré, Reserva Biológica Saltinho, Riacho da Sede, 21 September 2009, 08° 43′ 52.7″ S, 35°1 0′ 24.8″ W, 38 m a.s.l., Lima LRC, col (PML). One female imago and one female sub-imago with corresponding nymphal exuviae, same data except 22 September 2009 (DZRJ).
**Additional material.** One nymph, Brazil, Sergipe, Areia Branca, Estação Ecológica Serra de Itabaiana, Riacho Coqueiro, 17 March 2004, 10° 46′ 1.98″ S, 37° 20′ 21.6 W, 311 m a.s.l., Francischetti FC, col. Seven nymphs, Brazil, Sergipe, Areia Branca, Estação Ecológica Serra de Itabaiana, Riacho Água Fria, 18 March 2004, 10° 45′ 17.58″ S, 37° 20′ 31.5″ W, 292 m a.s.l., Francischetti FC, col. One nymph, Brazil, Sergipe, Areia Branca, Estação Ecológica Serra de Itabaiana, Rio dos Negros, 18 March 2004, 10° 44′ 49.2″ S, 37° 20′ 24.06″ W, 298 m a.s.l., Francischetti FC, col. Three nymphs, Brazil, Sergipe, Areia Branca, Estação Ecológica Serra de Itabaiana, Rio dos Negros, 18 March 2004, 10° 44′ 49.2″ S, 37° 20′ 24.06″ W, 298 m a.s.l., leaf litter, Francischetti FC, col. Same data, except thirty-three nymphs, marginal vegetation (CZNC).
**Life cycle association**
  Rearing adults from nymphs.

**Figure 31–32.**
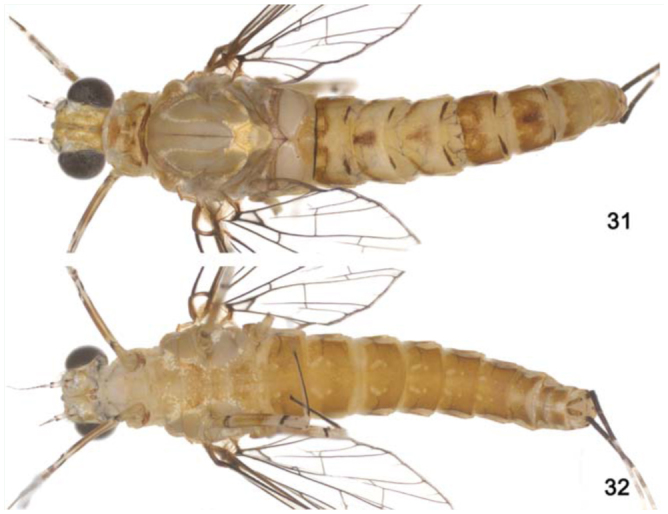
*Cloeodes redactus*, female adult. 31. d.v. 32. v.v. High quality figures are available online.

***Cloeodes redactus* Waltz and McCafferty, 1987 (Figure 1, 5, 6, 31, 32)**
Genus poss. *Cloeodes;* Roback 1966: pp. 133. *Cloeodes (Cloeodes) redactus* Waltz and McCafferty 1987a: pp. 204.
*Cloeodes redactus,* McCafferty and LugoOrtiz 1996: pp. 23; Domínguez et al. 2002: pp. 462; Domínguez et al. 2006: pp. 151; Nieto and Richard 2008: pp. 5; Nieto and Emmerich 2011: pp. 58.
**Diagnoses**
  **Nymphs:** 1) Labrum with dorsal arc of setae composed of 1 + 0 + 2 long, spine-like setae; 2) Segment III of labial palp rounded; 3) Forefemur apex projected, with two lanceolate setae; 4) Tarsal claw 0.3× length of tarsi; 5) General coloration of abdomen yellowishbrown with brown marks; 6) Spines on posterior margin of terga I absent; 7) Paraproct with 12 to 14 marginal spines; 8) Caudal filaments with posterior margin of segments with short spines on each segment and long spines on every four segments on cercus and every two segments on terminal filament.
**Adult: Female imago.** 1) Marginal intercalaries single; 2) Abdominal terga yellowishwhite, segments III, VI, and X with a red mark on medial region (Figure 31);
**Description**
  **Female imago**
  **Length.** *Body:* 5.0 mm; *antenna:* 0.8 mm; *fore-wing, tibia I, II, III, caudal filament:* broken.
**Head** (Figure 31). Yellowish-white. Compound eye black. Antenna yellowish-white, scape, and basal ¾ of pedicel white, apex of pedicel and flagellum washed with dark brown.
**Thorax** (Figure 31). Yellowish-white, median longitudinal suture brown. Apex of metascutellar protuberance dark brown.
*Anteronotal protuberance* rounded.
*Metascutellar protuberance* posteriorly pointed.
*Legs.* Yellowish-white. Femur washed with orange, with dark brown subapical transversal marking on anterior surface. Dorsal margin of tibia and tarsus dark brown.
*Fore-wing* hyaline, except opaque between C and R1. Longitudinal and cross veins dark brown. Marginal intercalary veins single; length of each intercalary vein between IMA and IMA2 0.3× distance between adjacent longitudinal veins.
*Hind wing* absent.
**Abdomen** (Figure 31). *Terga.* Yellowishwhite. Segments I–VI with a dark brown narrow transverse line on posterior margin, continuous only in segment I; segments III, VI and X with a red median marking; segments V, VI almost entirely washed with orange, segment VII washed with orange on anterior margin. Tracheation dark brown.
*Sterna* (Figure 32). Yellowish-white washed with orange, sigilla medioanterior and medioposterior lighter than background (Figure 32).
*Caudal filaments* yellowish-white with bases dark brown.
**Comments**
  The nymphs of *C. redactus*, like those of *C. barituensis*, lack spines on the posterior margin of tergum I. Among other characteristics, they can be differentiated based on abdominal color pattern (Figure 5, 6) and arrangement of spines on cerci. The female of *C. redactus*, described for the first time, is easily recognized by the presence of single marginal intercalary veins and color pattern (especially by the presence of anteromedian and posteromedian sigilla that are lighter than their background).
**Distribution** (Figure 1)
  COLOMBIA, HONDURAS, PERU, BRAZIL
  - Amazonas.
**Material examined**
  One nymph, Brazil, Amazonas, Manaus, PDBFF, BR 174, Fazenda Dimona, Igarapé (Rio Cuieiras) Capoeira Mista, 06 February 2001, 02° 20′ 98″ S, 60° 05′ 49″ W, Nessimian JL, col. (DZRJ). One female imago, Brazil, Amazonas, Manaus, Reserva Florestal Adolpho Ducke, Igarapé Barro Branco, 21 January 2010, 02° 53″ S, 59° 58′ W, Salles FF, Boldrini R and Cruz PV, col. (CZNC).
**Life cycle assocation**
  Rearing adults from nymphs.

**Figure 33–38.**
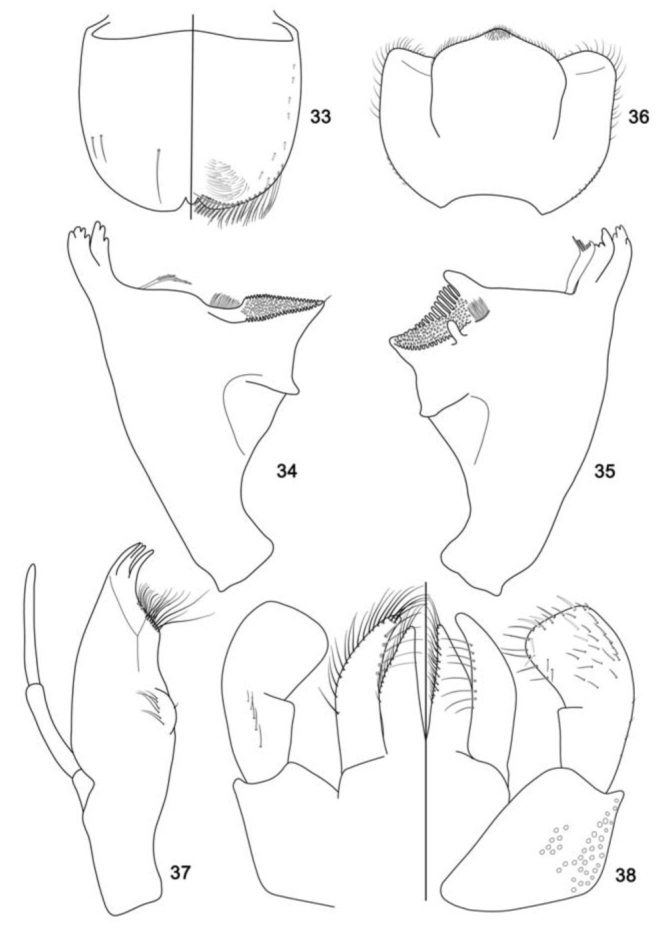
*Cloeodes spaceki*, sp. nov., nymph. 33. Labrum (left dorsal view, right ventral view). 34. Right mandible, ventral view. 35. Left mandible, dorsal view. 36. Hypopharynx, dorsal view. 37. Maxilla, ventral view. 38. Labium (left dorsal view, right ventral view). High quality figures are available online.

**Figure 39–42.**
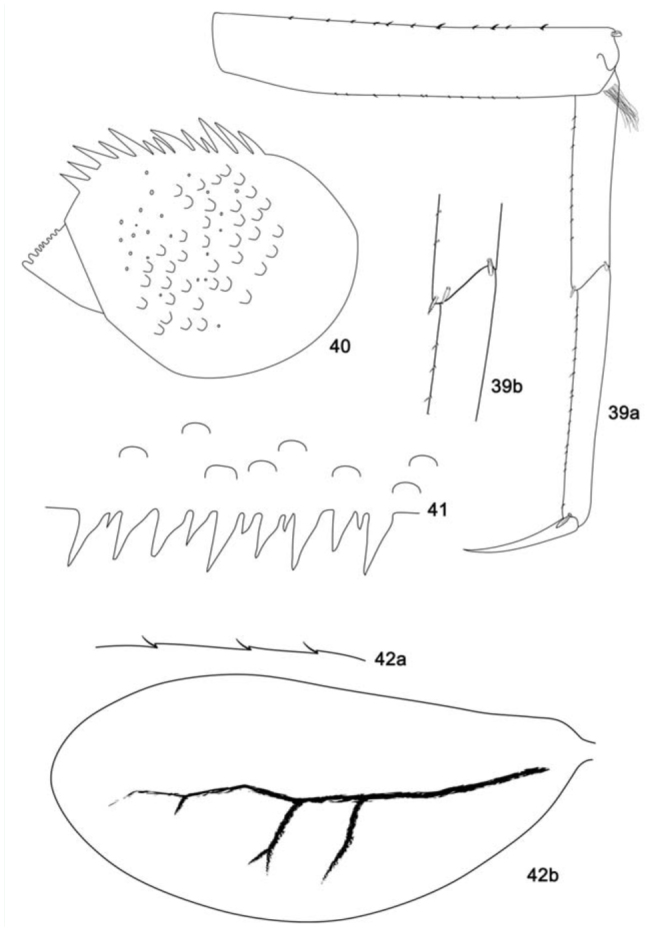
*Cloeodes spaceki*, sp. nov., nymph. 39. Fore-leg, anterior surface (a: general view; b: sub-tending bristle). 40. Paraproct (dorsal view). 41. Posterior margin of tergum IV. 42. Gill IV (a: details of gill margin; b: general view). High quality figures are available online.

***Cloeodes spaceki* Queiroz, Oliveira and Salles, sp. nov. (Figure 1, 7, 33–42)**
**Diagnoses**
  **Nymphs:** 1) Labrum with dorsal arc of setae composed of 1 + 0 + 2 long, spine-like setae (Figure 33); 2) Segment III of labial palp truncated (Figure 38); 3) Fore-femur apex with small projection, with two blunt setae (Figure 39a); 4) Tarsal claw 0.5× length of tarsi; 5) General coloration of abdomen yellowish-brown; segment I, anterior margin of segment II, and segment VI washed with dark brown (Figure 7); 6) Spines on posterior margin of terga I present; 7) Paraproct with 13 marginal spines (Figure 40); 8) Caudal filaments with posterior margin of segments with short spines on each segment and long spines on every two segments on cerci and terminal filament.
**Description**
  **Nymph**
  **Length.** *Body:* 5.2 mm; *cerci:* 1.7 mm; *terminal filament:* broken; *antenna:* broken.
**Head** (Figure 7). Coloration: brown.
*Antenna* light brown.
*Labrum* (Figure 33). Rectangular, broader than long; length about 0.7× maximum width; dorsal surface flat; distal margin with medial emargination and small process. Dorsally with few short, fine, simple setae scattered over surface; dorsal arc of setae composed of 1 + 0 + 2 long, spine-like setae; lateral margin bare. Ventrally with submarginal row of setae decreasing in length toward medial region, composed of lateral, anterolateral and medial setae bifurcated at middle and pectinate; ventral surface with seven to nine short, spine-like setae near lateral and anterolateral margin.
*Right mandible* (Figure 34). Inner and outer set of incisors with 3 and 5 denticles respectively. Prostheca slender, bifurcated at middle, inner lobe long, outer short, both frayed. Margin between prostheca and mola slightly convex; tuft of setae between prostheca and mola absent; tuft of spine-like setae at base of mola present; tuft of setae at apex of mola present, reduced to a bifid setae. Lateral margins almost straight; bare. Basal half bare.
*Left mandible* (Figure 35). Inner and outer set of incisors each with four denticles. Prostheca robust, apically denticulate and with combshape structure at apex. Margin between prostheca and mola straight, without crenulations; tuft of setae absent; tuft of spine-like setae at base of mola absent; subtriangular process wide, above level of area between prostheca and mola; denticles of mola not constricted; tuft of setae at apex of mola absent. Lateral margins almost straight; bare. Basal half bare.
*Hypopharynx* (Figure 36). Lingua subequal in length to superlingua; apex slightly convex; medial tuft of short setae present; distal half not expanded. Superlingua not expanded; fine, simple setae scattered over lateral and distal margin, and basal half of lateral margin with short, spine-like setae.
*Maxilla* (Figure 37). Crown of galea-lacinia with four denticles, inner dorsal row of setae with two pectinate denti-setae. Medial protuberance of galea with one short, spine-like setae and five long setae. Maxillary palp reaching apex of galea-lacinia; two-segmented; micropores scattered over surface; palp segment II 1.2× length of segment I; apex of last segment smooth.
*Labium* (Figure 38). Glossa basally broad, narrowing apically and subequal in length to paraglossa; inner margin with 18 long, spine-like setae increasing in length apically; outer margin with 13 long spine-like setae increasing in length distally; ventral surface with one row of seven simple setae near inner margin. Paraglossa sub-rectangular or straight, curved only at apex; apex with two rows of simple setae; outer margin with row of 14 long, spine-like setae; dorsally with a row of five long setae near inner margin; ventrally with a row of eight spine-like setae near inner margin. Labial palp with segment I 0.9× length of segments II and III combined; segment I covered with micropores; segment II with inner margin bare; outer margin with few, short, simple setae; dorsally with row of four or five spine-like, simple setae; segment III truncate; length 1.1× width; covered with long, spinelike, simple setae along margins and ventral surface.
**Thorax** (Figure 7). General coloration light brown with dark brown markings.
*Hind-wing pads* absent.
*Fore-leg* (Figure 39a). Yellowish-brown. Ratio of fore leg 1.2 : 0.6 : 0.7 : 0.3 mm.
*Fore-femur* (Figure 39a). Length about 4.0× maximum width; dorsally with row of 11 blunt setae (in lateral view they look like spine-like setae); length of setae about 0.1× maximum width of femur; apex with small projection; with two blunt setae; ventrally bare; anterior surface with micropores and scales.
*Tibia.* Dorsally bare; ventrally with row of 10 short, spine-like setae; anterior surface with scales scattered over surface; tibio-patelar suture present. Sub-tending bristle present (Figure 39b).
*Tarsus.* Dorsally bare; ventrally with one row of 16 spine-like setae and one lanceolate setae neat apex; tarsal claw bare, 0.5× length of tarsi.
*Mid- and hind-legs* similar to fore-leg.
**Abdomen** (Figure 7). General coloration yellowish-brown. Segment I, anterior margin of segment II, and segment VI washed with dark brown, remainder segments washed with brown, becoming darker toward lateral margin; segments I to VI with subtriangular dark brown mark laterally.
*Terga.* Surface with abundant scale-bases and micropores; posterior margin with long spines (2.1× longer than wide) often intercalated by short spines (2.0× longer than wide) (Figure 41); spines present in posterior margin of segments: I–X.
*Sterna.* Spines present in posterior margin of segments V–IX.
*Gill* (Figure 42a, b). Opaque; tracheae dark grey. Margin with narrow spines and short, fine, simple setae (Figure 42b). Tracheae extending from main trunk to inner margins.
*Paraproct* (Figure 40). With 13 marginal spines; surface with abundant scale-bases and micropores; posterolateral extension with blunt marginal spines.
*Caudal filaments.* Brown. Posterior margin of segments with short spines on each segment, and long spines on every two segments on cerci and terminal filament; inner margin of cercus and inner and outer margin of terminal filament with tufts of long, flat simple setae.
**Adult:** Unknown.
**Etymology**
  The specific epithet is in honor of Dr. Bruno Spacek Godoy, who collected the holotype.
**Comments**
  See comments under *C. maracatu*, sp. nov.
**Distribution** (Figure 1)
  BRAZIL - Goiás.
**Material examined**
  **Holotype.** Nymph (body in alcohol, mouthparts, legs, tergum IV, and paraprocts on three slides, mounting media Euparal®), Brazil, Goiás, Santa Isabel, Rio das Palmas, 19 November 2008, 15° 17′ 52.8″ S, 49° 20′ 39.1″ W, 580 m a.s.l., rock, sand, Godoy BS, col. (INPA).
**Paratypes.** Three nymphs, same data as holotype (DZRJ, IML, CZNC).

### Key to South American Species of Two-Winged *Cloeodes*

**Nymphs**
**1.** Segment III of labial palp truncated (Figure 38); tarsal claw equal or longer than 0.5× length of tarsi2
**1′.** Segment III of labial palp rounded (Figure 18); tarsal claw equal or less than 0.4× length of tarsi3
**2.** Fore-femur without apical projection; body color pattern as in Figure 2*C. auwe*
**2′**. Fore femur with a small apical projection (Figure 39a); body color pattern as in Figure 7*C. spaceki*, sp. nov.
**3.** Spines on posterior margin of segment I present*C. maracatu*, sp. nov.
**3′.** Spines on posterior margin of segment I absent4
**4.** Cercus with long spines on every four segments; body color pattern (Figure 5, 6)*C. redactus*
**4′.** Cercus with long spines on every two segments; body color pattern (Figure 3)*C. barituensis*

**Male imagines**
**1.** Fore-wing with marginal intercalary veins long, 0.9× distance between adjacent longitudinal veins (Figure 11)*C. auwe*
**1′.** Fore-wing with marginal intercalary veins short, less than 0.5× distance between adjacent longitudinal veins (as in Figure 28)2
**2.** Dorsal portion of turbinate eyes with inner margins divergent (Figure 25); thorax pale-yellow, medioscutelum with diagonal dark brown markings*C. maracatu*, sp. nov.
**2′.** Dorsal portion of turbinate eyes with margins parallel; thorax yellowish-brown without markings*C. barituensis*

## Abbreviations

CZNC,: Coleção Zoológica Norte Capixaba, Universidade Federal do Espírito Santo, São Mateus, Brazil
**DZRJ,**: Coleçāo Entomológica Prof. José Alfredo Pinheiro Dutra, Departamento de Zoologia, Universidade Federal do Rio de Janeiro, Rio de Janeiro, Brazil
**IML,**: Instituto Miguel Lillo, Tucumán, Argentina
**INPA,**: Invertebrate Collection of the Instituto Nacional de Pesquisas da Amazônia, Manaus, Brazil
